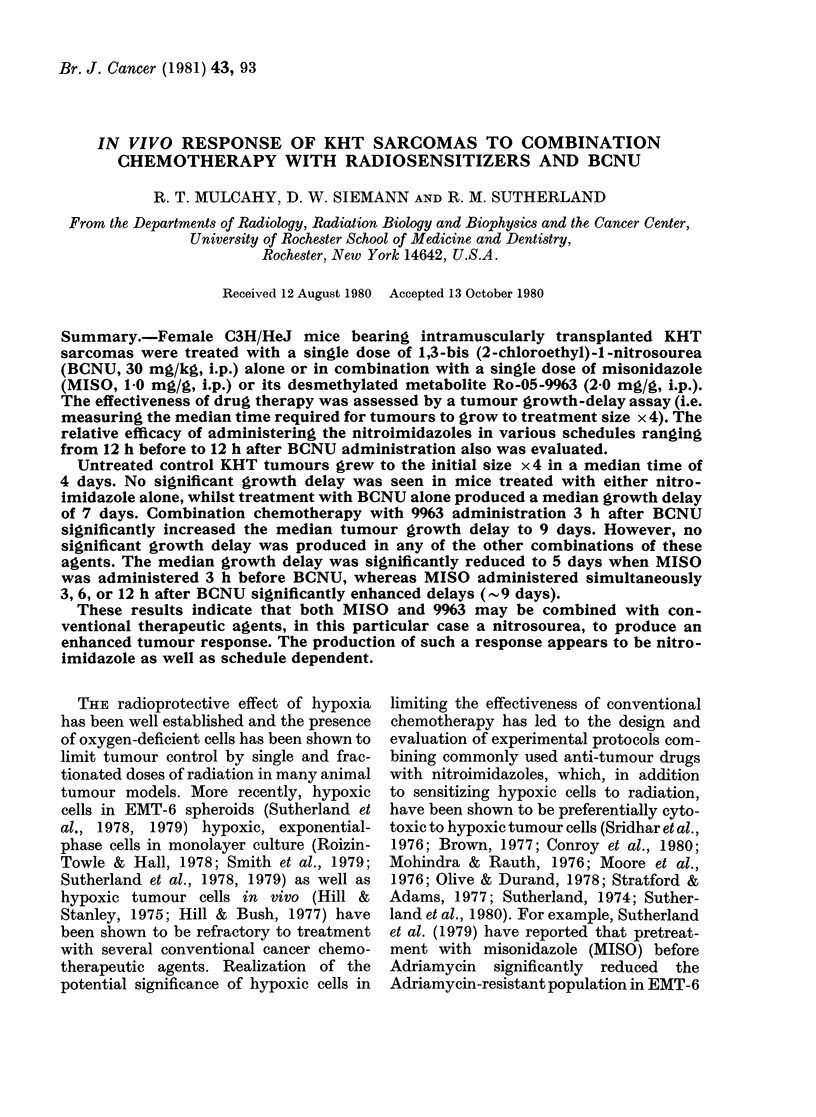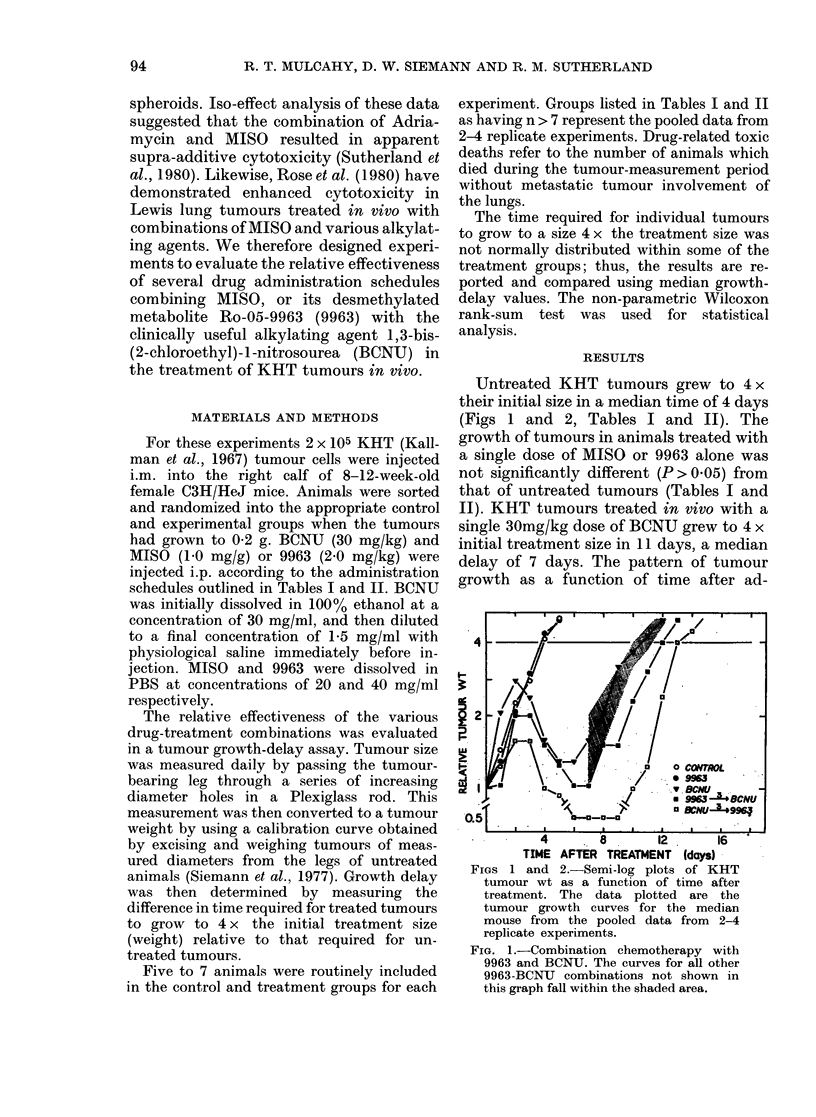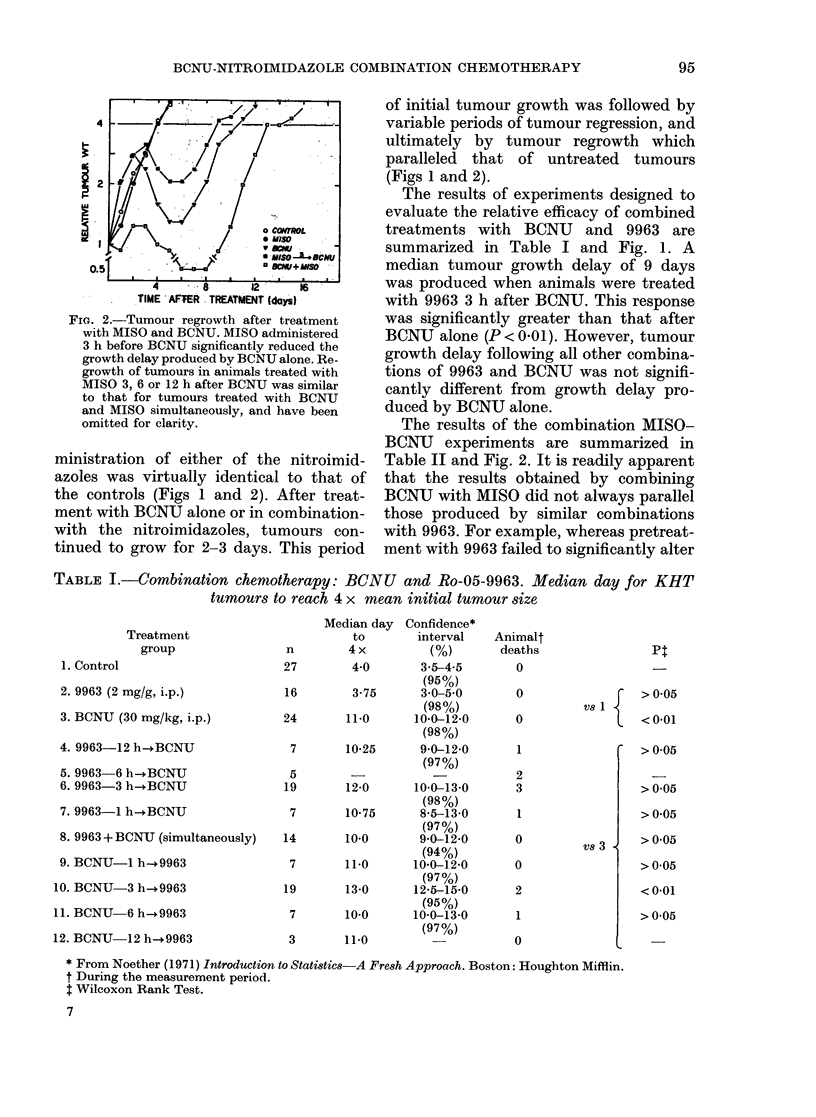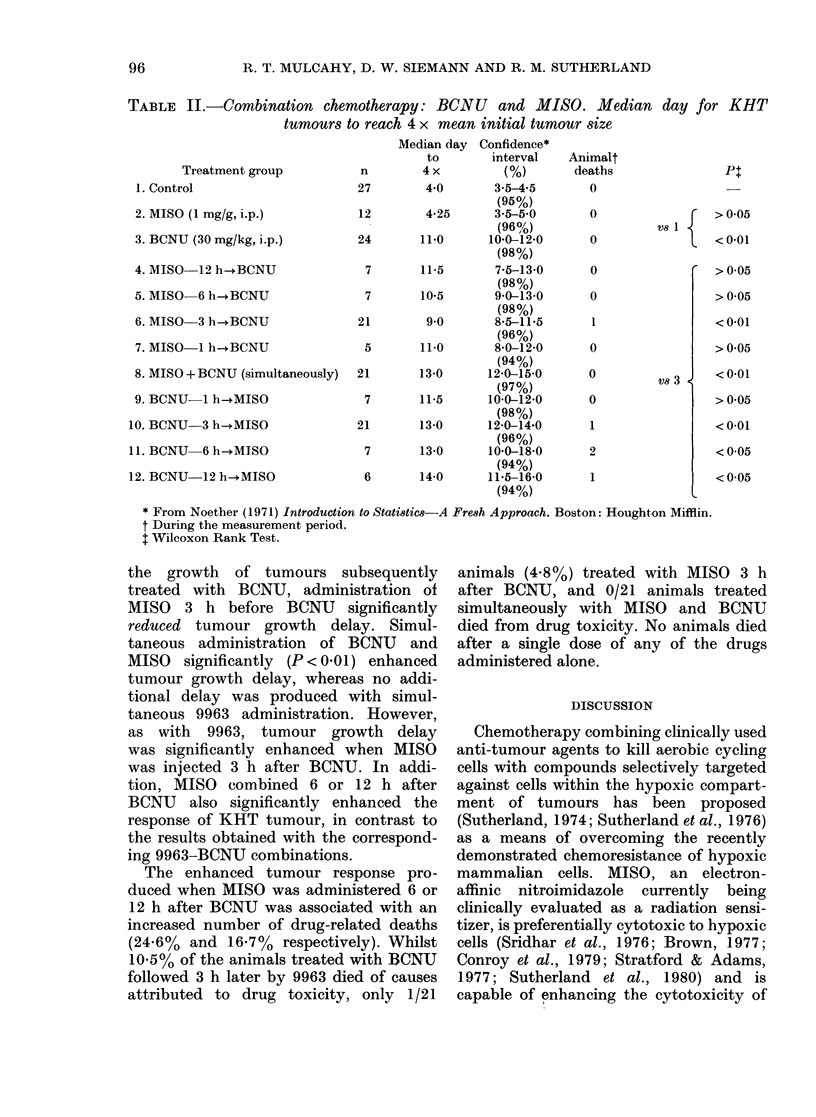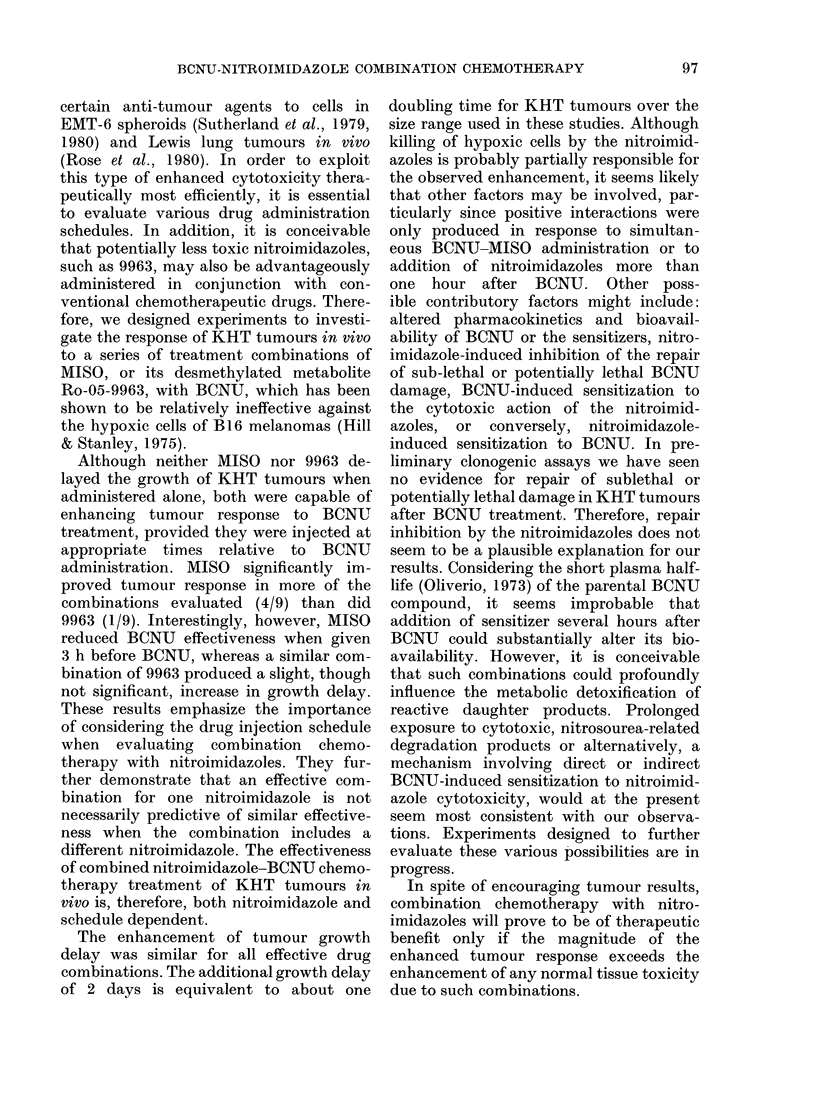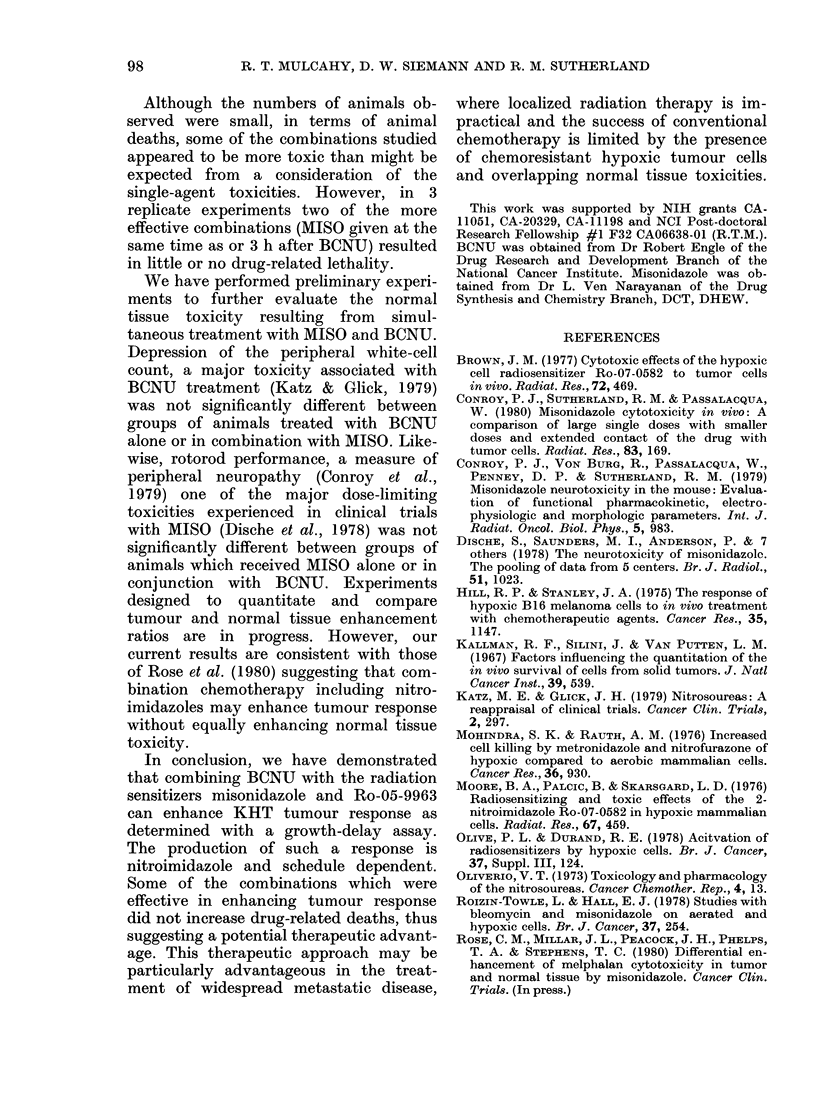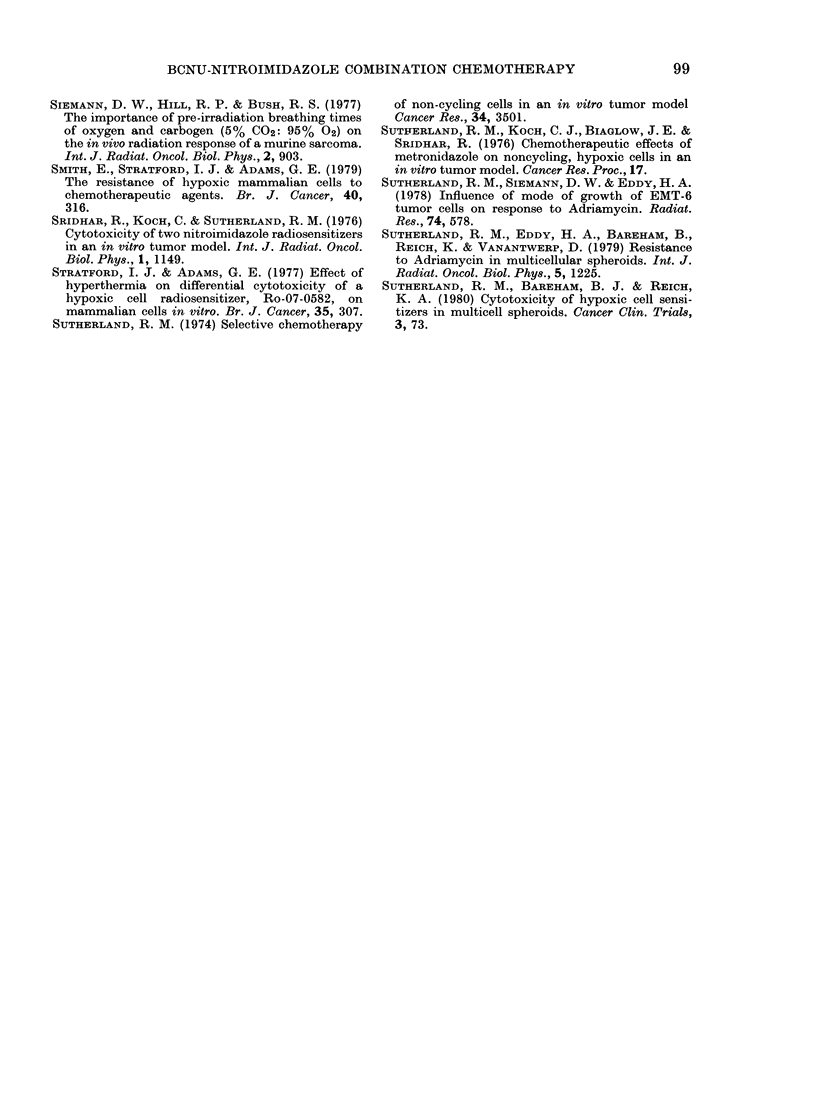# In vivo response of KHT sarcomas to combination chemotherapy with radiosensitizers and BCNU.

**DOI:** 10.1038/bjc.1981.13

**Published:** 1981-01

**Authors:** R. T. Mulcahy, D. W. Siemann, R. M. Sutherland

## Abstract

Female C3H/HeJ mice bearing intramuscularly transplanted KHT sarcomas were treated with a single dose of 1,3-bis (2-chloroethyl)-1-nitrosourea (BCNU, 30 mg/kg, i.p.) alone or in combination with a single dose of misonidazole (MISO, 1.0 mg/g, i.p.) or its desmethylated metabolite Ro-05-9963 (2.0 mg/g, i.p.). The effectiveness of drug therapy was assessed by a tumour growth-delay assay (i.e. measuring the median time required for tumours to grow to treatment size x 4). The relative efficacy of administering the nitroimidazoles in various schedules ranging from 12 h before to 12 h after BCNU administration also was evaluated. Untreated control KHT tumours grew to the initial size x 4 in a median time of 4 days. No significant growth delay was seen in mice treated with either nitroimidazole alone, whilst treatment with BCNU alone produced a median growth delay of 7 days. Combination chemotherapy with 9963 administration 3 h after BCNU significantly increased the median tumour growth delay to 9 days. However, no significant growth delay was produced in any of the other combinations of these agents. The median growth delay was significantly reduced to 5 days when MISO was administered 3 h before BCNU, whereas MISO administered simultaneously 3,6, or 12 h after BCNU significantly enhanced delays ( 9 days). These results indicate that both MISO and 0063 may be combined with conventional therapeutic agents, in this particular case a nitrosourea, to produce an enhanced tumour response. The production of such a response appears to be nitroimidazole as well as schedule dependent.


*Br. J. Cancer* (1981) **43**, 93

### *IN VIVO* RESPONSE OF KHT SARCOMAS TO COMBINATION CHEMOTHERAPY WITH RADIOSENSITIZERS AND BCNU

R. T. MULCAHY, D. W. SIEMANN AND R. M. SUTHERLAND

*From the Departments of Radiology, Radiation Biology and Biophysics and the Cancer Center, University of Rochester School of Medicine and Dentistry, Rochester, New York* 14642, *U.S.A.*

Received 12 August 1980 Accepted 13 October 1980

**Summary.—Female C3H/HeJ mice bearing intramuscularly transplanted KHT sarcomas were treated with a single dose of 1,3-bis (2-chloroethyl)-1-nitrosourea (BCNU, 30 mg/kg, i.p.) alone or in combination with a single dose of misonidazole (MISO, 1·0 mg/g, i.p.) or its desmethylated metabolite Ro-05-9963 (2·0 mg/g, i.p.). The effectiveness of drug therapy was assessed by a tumour growth-delay assay (i.e. measuring the median time required for tumours to grow to treatment size ×4). The relative efficacy of administering the nitroimidazoles in various schedules ranging from 12 h before to 12 h after BCNU administration also was evaluated.**

**Untreated control KHT tumours grew to the initial size ×4 in a median time of 4 days. No significant growth delay was seen in mice treated with either nitroimidazole alone, whilst treatment with BCNU alone produced a median growth delay of 7 days. Combination chemotherapy with 9963 administration 3 h after BCNU significantly increased the median tumour growth delay to 9 days. However, no significant growth delay was produced in any of the other combinations of these agents. The median growth delay was significantly reduced to 5 days when MISO was administered 3 h before BCNU, whereas MISO administered simultaneously 3, 6, or 12 h after BCNU significantly enhanced delays (~9 days).**

**These results indicate that both MISO and 9963 may be combined with conventional therapeutic agents, in this particular case a nitrosourea, to produce an enhanced tumour response. The production of such a response appears to be nitroimidazole as well as schedule dependent.**

THE radioprotective effect of hypoxia has been well established and the presence of oxygen-deficient cells has been shown to limit tumour control by single and fractionated doses of radiation in many animal tumour models. More recently, hypoxic cells in EMT-6 spheroids (Sutherland *et al.*, 1978, 1979) hypoxic, exponential-phase cells in monolayer culture (Roizin-Towle & Hall, 1978; Smith *et al.*, 1979; Sutherland *et al.*, 1978, 1979) as well as hypoxic tumour cells *in vivo* (Hill & Stanley, 1975; Hill & Bush, 1977) have been shown to be refractory to treatment with several conventional cancer chemotherapeutic agents. Realization of the potential significance of hypoxic cells in limiting the effectiveness of conventional chemotherapy has led to the design and evaluation of experimental protocols combining commonly used anti-tumour drugs with nitroimidazoles, which, in addition to sensitizing hypoxic cells to radiation, have been shown to be preferentially cytotoxic to hypoxic tumour cells (Sridhar *et al.*, 1976; Brown, 1977; Conroy *et al.*, 1980; Mohindra & Rauth, 1976; Moore *et al.*, 1976; Olive & Durand, 1978; Stratford & Adams, 1977; Sutherland, 1974; Sutherland *et al.*, 1980). For example, Sutherland *et al.* (1979) have reported that pretreatment with misonidazole (MISO) before Adriamycin significantly reduced the Adriamycin-resistant population in EMT-6

spheroids. Iso-effect analysis of these data suggested that the combination of Adriamycin and MISO resulted in apparent supra-additive cytotoxicity (Sutherland *et al.*, 1980). Likewise, Rose *et al.* (1980) have demonstrated enhanced cytotoxicity in Lewis lung tumours treated *in vivo* with combinations of MISO and various alkylating agents. We therefore designed experiments to evaluate the relative effectiveness of several drug administration schedules combining MISO, or its desmethylated metabolite Ro-05-9963 (9963) with the clinically useful alkylating agent 1,3-bis-(2-chloroethyl)-1-nitrosourea (BCNU) in the treatment of KHT tumours *in vivo*.

### MATERIALS AND METHODS

For these experiments $2 \times 10^5$ KHT (Kallman *et al.*, 1967) tumour cells were injected i.m. into the right calf of 8–12-week-old female C3H/HeJ mice. Animals were sorted and randomized into the appropriate control and experimental groups when the tumours had grown to 0·2 g. BCNU (30 mg/kg) and MISO (1·0 mg/g) or 9963 (2·0 mg/kg) were injected i.p. according to the administration schedules outlined in Tables I and II. BCNU was initially dissolved in 100% ethanol at a concentration of 30 mg/ml, and then diluted to a final concentration of 1·5 mg/ml with physiological saline immediately before injection. MISO and 9963 were dissolved in PBS at concentrations of 20 and 40 mg/ml respectively.

The relative effectiveness of the various drug-treatment combinations was evaluated in a tumour growth-delay assay. Tumour size was measured daily by passing the tumour-bearing leg through a series of increasing diameter holes in a Plexiglass rod. This measurement was then converted to a tumour weight by using a calibration curve obtained by excising and weighing tumours of measured diameters from the legs of untreated animals (Siemann *et al.*, 1977). Growth delay was then determined by measuring the difference in time required for treated tumours to grow to 4× the initial treatment size (weight) relative to that required for untreated tumours.

Five to 7 animals were routinely included in the control and treatment groups for each experiment. Groups listed in Tables I and II as having $n > 7$ represent the pooled data from 2–4 replicate experiments. Drug-related toxic deaths refer to the number of animals which died during the tumour-measurement period without metastatic tumour involvement of the lungs.

The time required for individual tumours to grow to a size 4× the treatment size was not normally distributed within some of the treatment groups; thus, the results are reported and compared using median growth-delay values. The non-parametric Wilcoxon rank-sum test was used for statistical analysis.

### RESULTS

Untreated KHT tumours grew to 4× their initial size in a median time of 4 days (Figs 1 and 2, Tables I and II). The growth of tumours in animals treated with a single dose of MISO or 9963 alone was not significantly different ($P > 0·05$) from that of untreated tumours (Tables I and II). KHT tumours treated *in vivo* with a single 30mg/kg dose of BCNU grew to 4× initial treatment size in 11 days, a median delay of 7 days. The pattern of tumour growth as a function of time after ad-

FIGS 1 and 2.—Semi-log plots of KHT tumour wt as a function of time after treatment. The data plotted are the tumour growth curves for the median mouse from the pooled data from 2–4 replicate experiments.

FIG. 1.—Combination chemotherapy with 9963 and BCNU. The curves for all other 9963-BCNU combinations not shown in this graph fall within the shaded area.

FIG. 2.—Tumour regrowth after treatment with MISO and BCNU. MISO administered 3 h before BCNU significantly reduced the growth delay produced by BCNU alone. Regrowth of tumours in animals treated with MISO 3, 6 or 12 h after BCNU was similar to that for tumours treated with BCNU and MISO simultaneously, and have been omitted for clarity.

ministration of either of the nitroimidazoles was virtually identical to that of the controls (Figs 1 and 2). After treatment with BCNU alone or in combination with the nitroimidazoles, tumours continued to grow for 2–3 days. This period of initial tumour growth was followed by variable periods of tumour regression, and ultimately by tumour regrowth which paralleled that of untreated tumours (Figs 1 and 2).

The results of experiments designed to evaluate the relative efficacy of combined treatments with BCNU and 9963 are summarized in Table I and Fig. 1. A median tumour growth delay of 9 days was produced when animals were treated with 9963 3 h after BCNU. This response was significantly greater than that after BCNU alone ($P < 0{\cdot}01$). However, tumour growth delay following all other combinations of 9963 and BCNU was not significantly different from growth delay produced by BCNU alone.

The results of the combination MISO–BCNU experiments are summarized in Table II and Fig. 2. It is readily apparent that the results obtained by combining BCNU with MISO did not always parallel those produced by similar combinations with 9963. For example, whereas pretreatment with 9963 failed to significantly alter

TABLE I.—*Combination chemotherapy: BCNU and Ro-05-9963. Median day for KHT tumours to reach* 4 × *mean initial tumour size*

| Treatment group | n | Median day to 4 × | Confidence* interval (%) | Animal† deaths | | P‡ |
|---|---|---|---|---|---|---|
| 1. Control | 27 | 4·0 | 3·5–4·5 (95%) | 0 | | — |
| 2. 9963 (2 mg/g, i.p.) | 16 | 3·75 | 3·0–5·0 (98%) | 0 | *vs* 1 | > 0·05 |
| 3. BCNU (30 mg/kg, i.p.) | 24 | 11·0 | 10·0–12·0 (98%) | 0 | *vs* 1 | < 0·01 |
| 4. 9963—12 h→BCNU | 7 | 10·25 | 9·0–12·0 (97%) | 1 | *vs* 3 | > 0·05 |
| 5. 9963—6 h→BCNU | 5 | — | — | 2 | *vs* 3 | — |
| 6. 9963—3 h→BCNU | 19 | 12·0 | 10·0–13·0 (98%) | 3 | *vs* 3 | > 0·05 |
| 7. 9963—1 h→BCNU | 7 | 10·75 | 8·5–13·0 (97%) | 1 | *vs* 3 | > 0·05 |
| 8. 9963 + BCNU (simultaneously) | 14 | 10·0 | 9·0–12·0 (94%) | 0 | *vs* 3 | > 0·05 |
| 9. BCNU—1 h→9963 | 7 | 11·0 | 10·0–12·0 (97%) | 0 | *vs* 3 | > 0·05 |
| 10. BCNU—3 h→9963 | 19 | 13·0 | 12·5–15·0 (95%) | 2 | *vs* 3 | < 0·01 |
| 11. BCNU—6 h→9963 | 7 | 10·0 | 10·0–13·0 (97%) | 1 | *vs* 3 | > 0·05 |
| 12. BCNU—12 h→9963 | 3 | 11·0 | — | 0 | *vs* 3 | — |

* From Noether (1971) *Introduction to Statistics—A Fresh Approach.* Boston: Houghton Mifflin.
† During the measurement period.
‡ Wilcoxon Rank Test.

TABLE II.—*Combination chemotherapy: BCNU and MISO. Median day for KHT tumours to reach* 4× *mean initial tumour size*

| Treatment group | n | Median day to 4× | Confidence* interval (%) | Animal† deaths | | P‡ |
|---|---|---|---|---|---|---|
| 1. Control | 27 | 4·0 | 3·5–4·5 (95%) | 0 | | — |
| 2. MISO (1 mg/g, i.p.) | 12 | 4·25 | 3·5–5·0 (96%) | 0 | vs 1 | > 0·05 |
| 3. BCNU (30 mg/kg, i.p.) | 24 | 11·0 | 10·0–12·0 (98%) | 0 | vs 1 | < 0·01 |
| 4. MISO—12 h→BCNU | 7 | 11·5 | 7·5–13·0 (98%) | 0 | vs 3 | > 0·05 |
| 5. MISO—6 h→BCNU | 7 | 10·5 | 9·0–13·0 (98%) | 0 | vs 3 | > 0·05 |
| 6. MISO—3 h→BCNU | 21 | 9·0 | 8·5–11·5 (96%) | 1 | vs 3 | < 0·01 |
| 7. MISO—1 h→BCNU | 5 | 11·0 | 8·0–12·0 (94%) | 0 | vs 3 | > 0·05 |
| 8. MISO + BCNU (simultaneously) | 21 | 13·0 | 12·0–15·0 (97%) | 0 | vs 3 | < 0·01 |
| 9. BCNU—1 h→MISO | 7 | 11·5 | 10·0–12·0 (98%) | 0 | vs 3 | > 0·05 |
| 10. BCNU—3 h→MISO | 21 | 13·0 | 12·0–14·0 (96%) | 1 | vs 3 | < 0·01 |
| 11. BCNU—6 h→MISO | 7 | 13·0 | 10·0–18·0 (94%) | 2 | vs 3 | < 0·05 |
| 12. BCNU—12 h→MISO | 6 | 14·0 | 11·5–16·0 (94%) | 1 | vs 3 | < 0·05 |

* From Noether (1971) *Introduction to Statistics—A Fresh Approach.* Boston: Houghton Mifflin.
† During the measurement period.
‡ Wilcoxon Rank Test.

the growth of tumours subsequently treated with BCNU, administration of MISO 3 h before BCNU significantly *reduced* tumour growth delay. Simultaneous administration of BCNU and MISO significantly ($P < 0{\cdot}01$) enhanced tumour growth delay, whereas no additional delay was produced with simultaneous 9963 administration. However, as with 9963, tumour growth delay was significantly enhanced when MISO was injected 3 h after BCNU. In addition, MISO combined 6 or 12 h after BCNU also significantly enhanced the response of KHT tumour, in contrast to the results obtained with the corresponding 9963–BCNU combinations.

The enhanced tumour response produced when MISO was administered 6 or 12 h after BCNU was associated with an increased number of drug-related deaths (24·6% and 16·7% respectively). Whilst 10·5% of the animals treated with BCNU followed 3 h later by 9963 died of causes attributed to drug toxicity, only 1/21 animals (4·8%) treated with MISO 3 h after BCNU, and 0/21 animals treated simultaneously with MISO and BCNU died from drug toxicity. No animals died after a single dose of any of the drugs administered alone.

### DISCUSSION

Chemotherapy combining clinically used anti-tumour agents to kill aerobic cycling cells with compounds selectively targeted against cells within the hypoxic compartment of tumours has been proposed (Sutherland, 1974; Sutherland *et al.*, 1976) as a means of overcoming the recently demonstrated chemoresistance of hypoxic mammalian cells. MISO, an electron-affinic nitroimidazole currently being clinically evaluated as a radiation sensitizer, is preferentially cytotoxic to hypoxic cells (Sridhar *et al.*, 1976; Brown, 1977; Conroy *et al.*, 1979; Stratford & Adams, 1977; Sutherland *et al.*, 1980) and is capable of enhancing the cytotoxicity of certain anti-tumour agents to cells in EMT-6 spheroids (Sutherland *et al.*, 1979, 1980) and Lewis lung tumours *in vivo* (Rose *et al.*, 1980). In order to exploit this type of enhanced cytotoxicity therapeutically most efficiently, it is essential to evaluate various drug administration schedules. In addition, it is conceivable that potentially less toxic nitroimidazoles, such as 9963, may also be advantageously administered in conjunction with conventional chemotherapeutic drugs. Therefore, we designed experiments to investigate the response of KHT tumours *in vivo* to a series of treatment combinations of MISO, or its desmethylated metabolite Ro-05-9963, with BCNU, which has been shown to be relatively ineffective against the hypoxic cells of B16 melanomas (Hill & Stanley, 1975).

Although neither MISO nor 9963 delayed the growth of KHT tumours when administered alone, both were capable of enhancing tumour response to BCNU treatment, provided they were injected at appropriate times relative to BCNU administration. MISO significantly improved tumour response in more of the combinations evaluated (4/9) than did 9963 (1/9). Interestingly, however, MISO reduced BCNU effectiveness when given 3 h before BCNU, whereas a similar combination of 9963 produced a slight, though not significant, increase in growth delay. These results emphasize the importance of considering the drug injection schedule when evaluating combination chemotherapy with nitroimidazoles. They further demonstrate that an effective combination for one nitroimidazole is not necessarily predictive of similar effectiveness when the combination includes a different nitroimidazole. The effectiveness of combined nitroimidazole–BCNU chemotherapy treatment of KHT tumours *in vivo* is, therefore, both nitroimidazole and schedule dependent.

The enhancement of tumour growth delay was similar for all effective drug combinations. The additional growth delay of 2 days is equivalent to about one doubling time for KHT tumours over the size range used in these studies. Although killing of hypoxic cells by the nitroimidazoles is probably partially responsible for the observed enhancement, it seems likely that other factors may be involved, particularly since positive interactions were only produced in response to simultaneous BCNU–MISO administration or to addition of nitroimidazoles more than one hour after BCNU. Other possible contributory factors might include: altered pharmacokinetics and bioavailability of BCNU or the sensitizers, nitroimidazole-induced inhibition of the repair of sub-lethal or potentially lethal BCNU damage, BCNU-induced sensitization to the cytotoxic action of the nitroimidazoles, or conversely, nitroimidazole-induced sensitization to BCNU. In preliminary clonogenic assays we have seen no evidence for repair of sublethal or potentially lethal damage in KHT tumours after BCNU treatment. Therefore, repair inhibition by the nitroimidazoles does not seem to be a plausible explanation for our results. Considering the short plasma half-life (Oliverio, 1973) of the parental BCNU compound, it seems improbable that addition of sensitizer several hours after BCNU could substantially alter its bioavailability. However, it is conceivable that such combinations could profoundly influence the metabolic detoxification of reactive daughter products. Prolonged exposure to cytotoxic, nitrosourea-related degradation products or alternatively, a mechanism involving direct or indirect BCNU-induced sensitization to nitroimidazole cytotoxicity, would at the present seem most consistent with our observations. Experiments designed to further evaluate these various possibilities are in progress.

In spite of encouraging tumour results, combination chemotherapy with nitroimidazoles will prove to be of therapeutic benefit only if the magnitude of the enhanced tumour response exceeds the enhancement of any normal tissue toxicity due to such combinations.

Although the numbers of animals observed were small, in terms of animal deaths, some of the combinations studied appeared to be more toxic than might be expected from a consideration of the single-agent toxicities. However, in 3 replicate experiments two of the more effective combinations (MISO given at the same time as or 3 h after BCNU) resulted in little or no drug-related lethality.

We have performed preliminary experiments to further evaluate the normal tissue toxicity resulting from simultaneous treatment with MISO and BCNU. Depression of the peripheral white-cell count, a major toxicity associated with BCNU treatment (Katz & Glick, 1979) was not significantly different between groups of animals treated with BCNU alone or in combination with MISO. Likewise, rotorod performance, a measure of peripheral neuropathy (Conroy *et al.*, 1979) one of the major dose-limiting toxicities experienced in clinical trials with MISO (Dische *et al.*, 1978) was not significantly different between groups of animals which received MISO alone or in conjunction with BCNU. Experiments designed to quantitate and compare tumour and normal tissue enhancement ratios are in progress. However, our current results are consistent with those of Rose *et al.* (1980) suggesting that combination chemotherapy including nitroimidazoles may enhance tumour response without equally enhancing normal tissue toxicity.

In conclusion, we have demonstrated that combining BCNU with the radiation sensitizers misonidazole and Ro-05-9963 can enhance KHT tumour response as determined with a growth-delay assay. The production of such a response is nitroimidazole and schedule dependent. Some of the combinations which were effective in enhancing tumour response did not increase drug-related deaths, thus suggesting a potential therapeutic advantage. This therapeutic approach may be particularly advantageous in the treatment of widespread metastatic disease, where localized radiation therapy is impractical and the success of conventional chemotherapy is limited by the presence of chemoresistant hypoxic tumour cells and overlapping normal tissue toxicities.

This work was supported by NIH grants CA-11051, CA-20329, CA-11198 and NCI Post-doctoral Research Fellowship #1 F32 CA06638-01 (R.T.M.). BCNU was obtained from Dr Robert Engle of the Drug Research and Development Branch of the National Cancer Institute. Misonidazole was obtained from Dr L. Ven Narayanan of the Drug Synthesis and Chemistry Branch, DCT, DHEW.

### REFERENCES

Brown, J. M. (1977) Cytotoxic effects of the hypoxic cell radiosensitizer Ro-07-0582 to tumor cells *in vivo*. *Radiat. Res.*, **72**, 469.

Conroy, P. J., Sutherland, R. M. & Passalacqua, W. (1980) Misonidazole cytotoxicity *in vivo*: A comparison of large single doses with smaller doses and extended contact of the drug with tumor cells. *Radiat. Res.*, **83**, 169.

Conroy, P. J., Von Burg, R., Passalacqua, W., Penney, D. P. & Sutherland, R. M. (1979) Misonidazole neurotoxicity in the mouse: Evaluation of functional pharmacokinetic, electrophysiologic and morphologic parameters. *Int. J. Radiat. Oncol. Biol. Phys.*, **5**, 983.

Dische, S., Saunders, M. I., Anderson, P. & 7 others (1978) The neurotoxicity of misonidazole. The pooling of data from 5 centers. *Br. J. Radiol.*, **51**, 1023.

Hill, R. P. & Stanley, J. A. (1975) The response of hypoxic B16 melanoma cells to *in vivo* treatment with chemotherapeutic agents. *Cancer Res.*, **35**, 1147.

Kallman, R. F., Silini, J. & Van Putten, L. M. (1967) Factors influencing the quantitation of the *in vivo* survival of cells from solid tumors. *J. Natl Cancer Inst.*, **39**, 539.

Katz, M. E. & Glick, J. H. (1979) Nitrosoureas: A reappraisal of clinical trials. *Cancer Clin. Trials*, **2**, 297.

Mohindra, S. K. & Rauth, A. M. (1976) Increased cell killing by metronidazole and nitrofurazone of hypoxic compared to aerobic mammalian cells. *Cancer Res.*, **36**, 930.

Moore, B. A., Palcic, B. & Skarsgard, L. D. (1976) Radiosensitizing and toxic effects of the 2-nitroimidazole Ro-07-0582 in hypoxic mammalian cells. *Radiat. Res.*, **67**, 459.

Olive, P. L. & Durand, R. E. (1978) Acitvation of radiosensitizers by hypoxic cells. *Br. J. Cancer*, **37**, Suppl. III, 124.

Oliverio, V. T. (1973) Toxicology and pharmacology of the nitrosoureas. *Cancer Chemother. Rep.*, **4**, 13.

Roizin-Towle, L. & Hall, E. J. (1978) Studies with bleomycin and misonidazole on aerated and hypoxic cells. *Br. J. Cancer*, **37**, 254.

Rose, C. M., Millar, J. L., Peacock, J. H., Phelps, T. A. & Stephens, T. C. (1980) Differential enhancement of melphalan cytotoxicity in tumor and normal tissue by misonidazole. *Cancer Clin. Trials*. (In press.)

SIEMANN, D. W., HILL, R. P. & BUSH, R. S. (1977) The importance of pre-irradiation breathing times of oxygen and carbogen (5% $CO_2$: 95% $O_2$) on the *in vivo* radiation response of a murine sarcoma. *Int. J. Radiat. Oncol. Biol. Phys.*, **2**, 903.

SMITH, E., STRATFORD, I. J. & ADAMS, G. E. (1979) The resistance of hypoxic mammalian cells to chemotherapeutic agents. *Br. J. Cancer*, **40**, 316.

SRIDHAR, R., KOCH, C. & SUTHERLAND, R. M. (1976) Cytotoxicity of two nitroimidazole radiosensitizers in an *in vitro* tumor model. *Int. J. Radiat. Oncol. Biol. Phys.*, **1**, 1149.

STRATFORD, I. J. & ADAMS, G. E. (1977) Effect of hyperthermia on differential cytotoxicity of a hypoxic cell radiosensitizer, Ro-07-0582, on mammalian cells *in vitro*. *Br. J. Cancer*, **35**, 307.

SUTHERLAND, R. M. (1974) Selective chemotherapy of non-cycling cells in an *in vitro* tumor model *Cancer Res.*, **34**, 3501.

SUTHERLAND, R. M., KOCH, C. J., BIAGLOW, J. E. & SRIDHAR, R. (1976) Chemotherapeutic effects of metronidazole on noncycling, hypoxic cells in an *in vitro* tumor model. *Cancer Res. Proc.*, **17**.

SUTHERLAND, R. M., SIEMANN, D. W. & EDDY, H. A. (1978) Influence of mode of growth of EMT-6 tumor cells on response to Adriamycin. *Radiat. Res.*, **74**, 578.

SUTHERLAND, R. M., EDDY, H. A., BAREHAM, B., REICH, K. & VANANTWERP, D. (1979) Resistance to Adriamycin in multicellular spheroids. *Int. J. Radiat. Oncol. Biol. Phys.*, **5**, 1225.

SUTHERLAND, R. M., BAREHAM, B. J. & REICH, K. A. (1980) Cytotoxicity of hypoxic cell sensitizers in multicell spheroids. *Cancer Clin. Trials*, **3**, 73.